# A Real-Time Flame Detection Method Using Deformable Object Detection and Time Sequence Analysis

**DOI:** 10.3390/s23208616

**Published:** 2023-10-21

**Authors:** Jingyuan Zhang, Bo Shi, Bin Chen, Heping Chen, Wangming Xu

**Affiliations:** 1 School of Information Science and Engineering, Wuhan University of Science and Technology, Wuhan 430081, China; bada83@163.com (J.Z.); chenbin@wust.edu.cn (B.C.); 2 Sureland Industrial Fire Safety Limited, Beijing 101300, China; shi4bo8@outlook.com; 3 Engineering Research Center for Metallurgical Automation and Detecting Technology of Ministry of Education, Wuhan University of Science and Technology, Wuhan 430081, China

**Keywords:** flame detection, YOLOv5, deformable convolution, Focal Loss, EIOU Loss, time sequence analysis

## Abstract

Timely and accurate flame detection is a very important and practical technology for preventing the occurrence of fire accidents effectively. However, the current methods of flame detection are still faced with many challenges in video surveillance scenarios due to issues such as varying flame shapes, imbalanced samples, and interference from flame-like objects. In this work, a real-time flame detection method based on deformable object detection and time sequence analysis is proposed to address these issues. Firstly, based on the existing single-stage object detection network YOLOv5s, the network structure is improved by introducing deformable convolution to enhance the feature extraction ability for irregularly shaped flames. Secondly, the loss function is improved by using Focal Loss as the classification loss function to solve the problems of the imbalance of positive (flames) and negative (background) samples, as well as the imbalance of easy and hard samples, and by using EIOU Loss as the regression loss function to solve the problems of a slow convergence speed and inaccurate regression position in network training. Finally, a time sequence analysis strategy is adopted to comprehensively analyze the flame detection results of the current frame and historical frames in the surveillance video, alleviating false alarms caused by flame shape changes, flame occlusion, and flame-like interference. The experimental results indicate that the average precision (AP) and the F-Measure index of flame detection using the proposed method reach 93.0% and 89.6%, respectively, both of which are superior to the compared methods, and the detection speed is 24–26 FPS, meeting the real-time requirements of video flame detection.

## 1. Introduction

Fire disasters are one of the most common and dangerous types of disasters, characterized by a low predictability and rapid spread. Once a fire disaster occurs, it can cause significant property damage, disrupt the ecological balance, and even endanger lives. Rapid and accurate detection and identification in the early stages of a fire disaster are crucial for fire safety and prevention. Traditional fire detection methods mainly rely on smoke detectors, heat detectors, infrared detectors, and other devices [1]. These detectors are widely used due to their low cost and ease of use. However, smoke detectors and heat detectors are limited to small enclosed spaces, and smoke detectors are prone to false alarms in areas with a high dust concentration. Infrared detectors are mainly used in large open spaces, but they cannot determine the type of high-temperature objects and can be affected by high-temperature equipment, leading to false alarms. With the rapid development of computer vision and the widespread use of cameras in the field of security, flame detection based on image/video surveillance has emerged as a new approach to fire detection [2,3]. Compared to traditional detectors, image/video-based flame detection techniques offer advantages such as a fast detection speed, strong applicability, and the ability to quantify flame position and size.

Traditional flame image-processing techniques typically rely on factors such as color, texture, and gradients of the flames in the image [4,5] for feature design. They may also employ clustering methods [6] to extract features from the target regions and utilize classifiers such as Support Vector Machines [7], differential methods [8], or XGBoost classifiers [9] for flame classification and recognition. However, these detection methods are highly susceptible to the influence of external environmental factors, leading to a significant number of false positives and false negatives.

In recent years, object detection methods based on deep learning have rapidly advanced, showing significant improvements in detection accuracy and efficiency compared to traditional image-processing methods. Among the various object detection methods, there are two main categories: two-stage detection methods, primarily based on R-CNN series networks, and single-stage detection methods, primarily based on the YOLO series, RetinaNet, and SSD, etc. Two-stage detection methods first propose candidate bounding boxes and then classify them, offering advantages in terms of accuracy and localization precision. Single-stage detection methods directly predict the bounding boxes of objects in an image, providing simpler structures and faster speeds. CHAO et al. [10] proposed a global information-guided flame detection method using Faster R-CNN, but it had a large number of parameters and slow detection speed. ZHAO et al. [11] proposed the Fire-YOLO model based on YOLOv3, which achieved better results but still had room for improvement in terms of accuracy. JIANG et al. [12] utilized the RetinaNet model for flame detection but encountered issues of sparse flame omissions. LU et al. [13] presented the SSD_MobileNet model by modifying the VGG16 network in the classic SSD and incorporating depth-wise separable convolutions, which reduced flame omissions but still resulted in a significant number of false positives.

The lightweight single-stage object detection network is known for its flexibility, speed, and ease of use, and it is widely used in current object detection algorithms. However, directly applying it to flame detection tasks does not yield satisfactory results. This is because flame objects, unlike general rigid objects, often exhibit irregular shapes, diverse textures and edges, and imbalances in positive and negative samples and in easy and hard samples. In our previous experiments using the popular YOLOv5s network on images and videos containing flame objects, the flame recognition rate was found to be low, with many instances of missed real flames and false positives for flame-likes, making it unsuitable for practical surveillance systems.

Therefore, to address these challenges and improve the accuracy of flame detection in video surveillance scenarios, we propose a real-time flame detection method based on deformable object detection and time sequence analysis. Firstly, to better extract flame features, we introduce an improved deformable convolutional network (DCN) [14,15] that can handle the irregular and diverse shapes of flame objects. Secondly, to tackle the issue in flame detection of imbalances in positive and negative samples and in easy and hard samples, we improve the loss functions by employing Focal Loss [16] as the classification loss function to alleviate the problem of sample imbalance. Additionally, we utilize EIOU Loss [17] as the regression loss function to address the slow convergence speed of GIOU Loss [18] and enhance the detection performance. Lastly, considering the temporal characteristics of surveillance videos, we adopt a time sequence analysis strategy to mitigate the effects of flame disturbance deformation, occlusion, and false alarms caused by suspected flame objects in a single frame, thus reducing false positives. The experimental results demonstrate that the proposed method not only improves the accuracy and recall rate of flame detection, but also achieves a real-time detection speed, which is beneficial for fire prevention.

## 2. Methods

In the context of video surveillance, the primary task of flame detection is to continuously monitor the video information captured by cameras in a surveillance scene and make reasonable judgments about the presence of flame objects in that scene. When a real flame object is detected, it promptly sends out an alarm notification.

The task of flame object detection differs from usual rigid object detection tasks due to challenges such as variations in flame object shapes, imbalances in positive and negative samples and in easy and hard samples, occlusion by foreign objects, and confusion with potential flame-like objects. Therefore, it is very crucial to accurately detect flame objects in individual frames of surveillance videos. Additionally, the effective use of time sequence analysis strategies to comprehensively analyze detection results across video frame sequences can help to determine the presence of real fire objects in the current surveillance scene, thereby reducing false alarm rates.

**Figure 1:**
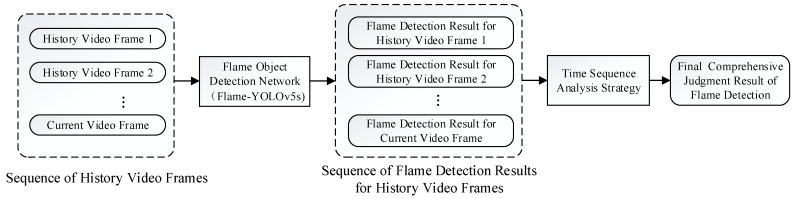
Flowchart of the proposed flame detection method.

The workflow of the proposed flame detection method in this paper is illustrated in Figure 1. An improved flame object detection network, namely Flame-YOLOv5s, processes each frame of the surveillance video to obtain a time sequence of the detection results. Then, based on our designed time sequence analysis strategy, it comprehensively analyzes the flame detection results, including that of the current frame, to determine the presence of real flame objects in the current moment of the surveillance scene. The two key aspects of this method are the improvement strategies of the flame object detection network Flame-YOLOv5s and the time sequence analysis strategy for video frames, which will be explained in detail below.

### 2.1. Network for Flame Object Detection

In the context of flame object detection in surveillance videos, the key step is to determine whether there is a real flame object in each frame of the video. Generally, existing object detection networks can be used for this purpose. However, considering the real-time requirements of video surveillance, we focus on improving a lightweight single-stage object detection network. In the commonly used Convolutional Neural Network (CNN) structure, the convolutional kernels typically sample the input feature maps at fixed positions and the pooling layers gradually reduce the feature map size. The ROI pooling layer generates spatially constrained regions of interest (ROI). However, these approaches cause some issues. For example, the fixed weights of the convolutional kernels result in the same receptive field size when processing the different regions of an image, which is unreasonable for deep convolutional neural networks that encode positional information. Different positions may correspond to objects with different scales or deformations, requiring methods that can automatically adjust the scale or receptive field. Another issue is that the effectiveness of object detection heavily relies on feature-based bounding boxes, which is not optimal, especially for non-grid objects.

Due to the irregularity and diversity of flame shapes, ordinary convolutions have a limited adaptability to extract flame features. To more effectively extract flame features, Deformable Convolution (DCN) [14] is introduced in our method to improve the backbone network of the object detection network.

By introducing learnable offsets to the convolutional kernel, DCN aims to concentrate the feature points in the regions of interest, thereby enhancing the modeling capability for unknown shape transformations, which can be expressed in the following formula:

(1) $$y(p)=\sum_{k=1}^{K}w_{k}x\left(p+p_{k}+\Delta_{p_{k}}\right)$$

In the given formula, x and y are the input feature map and the output feature map, respectively, and p denotes an arbitrary location on a feature map. *K* is the number of samples for a given convolutional kernel. The weight and pre-defined offset for the *k*-th position are denoted as $w_{k}$ and *p_k_* respectively, and $\Delta_{p_{k}}$ denotes the offset that is to be learned for the *k*-th position.

DCNv2 [15] further introduces an adjustment mechanism to alleviate the issues caused by either too small or too large offsets, which can result in sample points being unrelated to the object. Through this adjustment mechanism, it can adjust not only the offset of the sample points, but also the weights of the sample points. This can be expressed in the following formula:

(2) $$y(p)=\sum_{k=1}^{K}w_{k}x\left(p+p_{k}+\Delta_{p_{k}}\right)\Delta_{m_{k}}$$

In the given formula, $\Delta_{m_{k}}$ denotes the learnable adjustment scalar for the *k*-th position, which ranges between 0 and 1.

In this paper, we aim to improve the YOLOv5s object detection network for a better suitability to flame detection tasks. The modified network is referred to as Flame-YOLOv5s. In the original YOLOv5s network, there are four CBL structures in the backbone, which stands for the structure of “CNN + BN + LeakyReLu”. In this paper, the standard convolutional layers (CNN) within these structures are replaced with improved deformable convolutions (DCNv2), and the original LeakyReLu activation function is replaced with the SiLU activation function. This results in the formation of the DBS structure, which stands for the structure of “DCNv2 + BN + SiLU”. The SiLU function has a smoother curve near zero than the LeakyReLu function, and its output ranges between 0 and 1 because it uses the sigmoid function, which makes SiLU perform better than LeakyReLu.

**Figure 2:**
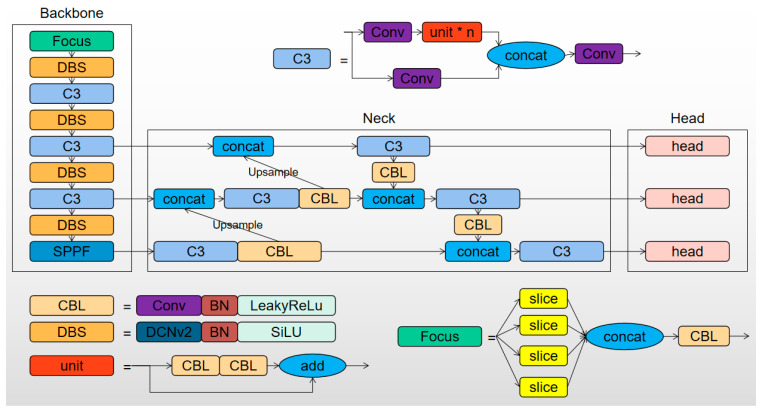
Overall network architecture of Flame-YOLOv5s.

Therefore, the overall architecture of the Flame-YOLOv5s network is shown in Figure 2, consisting of three components: the backbone, neck, and head. The backbone is used for extracting object features, which consists of the Focus, DBS, C3, and SPPF structures. The neck component adopts the “FPN + PAN” structure, which is primarily used for enhancing the extracted features. The head component utilizes three detection heads that perform downsampling 8, 16, and 32 times on the original image to generate feature vectors of different sizes, which is used for detecting objects of different scales.

### 2.2. Loss Functions

In the task of flame detection, the total loss of model training consists of two parts: classification loss and regression loss.

When using single-stage object detection algorithms like YOLOv5s to detect flame objects from input images, a large number of anchor boxes are generated to locate the flames. However, even if a real flame object (positive sample) is present in an image, its area or quantity is relatively small compared to the background (negative sample), because there are a large number of anchor boxes located in the background region. This leads to a severe imbalance between positive and negative samples.

In contrast, two-stage object detection methods such as R-CNN only generate candidate boxes in the first stage using the Region Proposal Network (RPN). These methods simply distinguish between the background and the foreground without distinguishing specific classes, thereby filtering out a significant portion of anchor boxes belonging to the background. While this approach alleviates the sample imbalance problem to some extent, it does not fully address the issue. In the second stage, heuristic sampling or OHEM (Online Hard Example Mining) is often employed to further alleviate the sample imbalance problem. On the other hand, single-stage methods sacrifice the generation of candidate boxes to improve detection speed, directly performing more challenging fine-grained classification on anchor boxes, which lacks a filtering process for the anchor boxes.

In flame detection task, the imbalance between positive and negative samples makes flame localization and classification more challenging. At the same time, some ambiguous flame-like regions may exhibit low discriminative features, making them be the hard samples. To address these issues and train the improved Flame-YOLOv5s network for flame detection, we adopt Focal Loss as the classification loss function. By focusing on the difficulty of classification, the Focal Loss assigns more importance to hard examples, thereby mitigating the imbalance between flame and background samples and improving the overall performance of the model.

Focal Loss is defined as an extension of the *BCE* (Binary Cross-Entropy) loss function

(3) $$BCE(p_{t})=-\alpha_{t}\ln(p_{t})$$

that introduces a modulation factor to reduce the weight of easily classified samples and focus on training hard samples. Mathematically, the Focal Loss can be defined as follows:

(4) $$FL(p_{t})=-\alpha_{t}{\left(1-p_{t}\right)}^{\gamma }\ln(p_{t})$$

Parameter $\alpha_{t}$ is a sample weight factor used to balance positive and negative samples, parameter γ≥0 serves as an adjustable focusing parameter, and parameter $p_{t}$ reflects the proximity to the class. As observed, Focal Loss not only addresses the issue of imbalance between positive and negative samples, but also tackles the problem of imbalance between easy and hard samples. Therefore, Focal Loss is well-suited for flame object detection tasks, as it effectively handles both types of sample imbalance.

In addition, the original YOLOv5s network utilized the GIOU (Generalized Intersection over Union) Loss function for regression, which addresses the gradient issue arising from non-overlapping predicted and ground truth boxes. GIOU inherits all the advantages of IOU but still has drawbacks such as slow convergence and imprecise regression due to its excessive reliance on the intersection over union ratio. To enhance the convergence speed, improve the flame recognition accuracy, and achieve more precise localization in the flame detection model, we adopt the EIOU (Efficient Intersection over Union) Loss [16] for calculating the regression loss of the bounding boxes. The EIOU Loss aims to address the limitations of the GIOU Loss, leading to faster convergence and more accurate regression in flame detection.

EIOU takes into account the overlapping region of box regression, the distance between the center points, and the aspect ratio, achieving fast convergence. The EIOU Loss can be represented as follows:

(5) $$L_{EIOU}=L_{IOU}+L_{dis}+L_{asp}=1-IOU+\frac{\rho^{2}(b,b^{gt})}{c^{2}}+\frac{\rho^{2}(w,w^{gt})}{c_{w}{}^{2}}+\frac{\rho^{2}(h,h^{gt})}{c_{h}{}^{2}}$$

**Figure 3:**
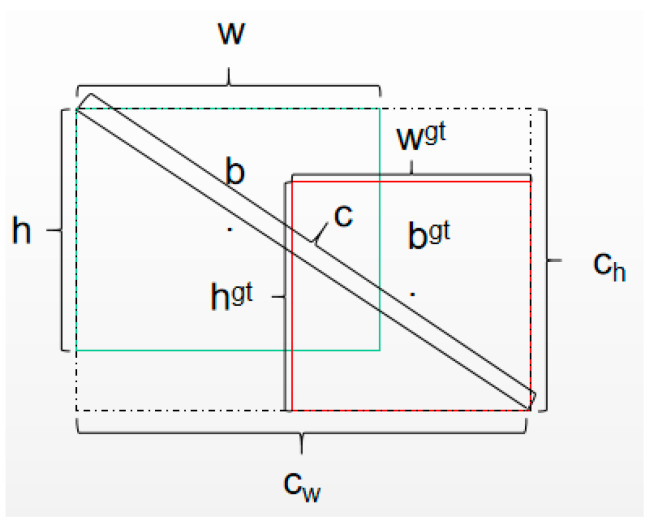
Parameter diagram of EIOU.

In above equation, $\rho^{2}(b,b^{gt})$ represent the Euclidean distance between the center points of the predicted box and the ground truth box, $\rho^{2}(w,w^{gt})$ represents the squared difference in width, and $\rho^{2}(h,h^{gt})$ represents the squared difference in height. On the other hand, $c_{w}$, $c_{h}$, and c represent the width, height, and diagonal distance of the minimum enclosing region of the predicted box and the ground truth box, respectively. The parameter information of EIOU is depicted in Figure 3.

### 2.3. Time Sequence Analysis Strategy

When dealing with the flame video, we process every image frame using the proposed Flame-YOLOv5s network. Despite the improved performance obtained due to the modification of the object detection network, it is inevitable to encounter the missed detection of flame objects due to flame disturbance deformation or partial occlusion. There may also be false detections of flame objects caused by occasional high confidence in flame-like objects. However, it has been observed through experiments that such missed detections and false detections occur with a low frequency or for short durations. Therefore, leveraging the temporal characteristics of video data, a time sequence analysis strategy can be employed to eliminate the impact of these missed detections and false detections on the final determination of the presence of flame objects.

**Figure 4:**
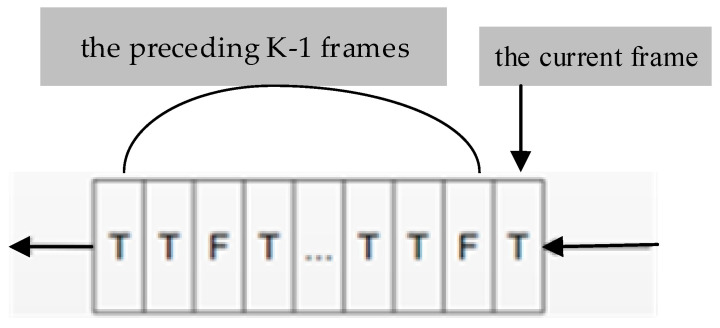
Time sequence of flame detection results for video frames.

In this paper, a time sequence is designed by incorporating temporal information from the preceding and subsequent frames of a video. The queue has a window size of K and is used to store the detection results of the current frame and the preceding K-1 frames. The detection results are represented by T (indicating the presence of flame in the frame) or F (indicating the absence of flame in the frame). The time sequence is illustrated in Figure 4.

The employed strategies for analyzing temporal information are as follows:

(1) Continuous Counting Strategy: If the number of consecutive frames with detected flame objects in the time sequence exceeds a specified threshold, it is considered that a real flame exists in the monitored video and an alarm signal is triggered.
(2) Cumulative Counting Strategy: When the time sequence is full, the number of frames with detected flame objects is counted, represented as n. If the percentage of frames with detected flame objects $\frac{n}{K}\times 100\%$ exceeds a specified threshold τ, it is considered that a real flame exists in the monitored video and an alarm signal is triggered.
(3) Alarm Termination Strategy: Both of the above strategies are applied simultaneously. When either of them are satisfied, an alarm is triggered. If the time sequence is full and the percentage of frames with detected flame objects $\frac{n}{K}\times 100\%$ is less than a specified threshold τ, it is considered that the alarm has ended and the system will wait for the next alarm trigger.

## 3. Experiments and Results

The experimental environment in this study consisted of a Windows 10 operating system and an NVIDIA GeForce RTX 3080 GPU. All the experiments were implemented using the deep learning framework PyTorch. The main parameters for the model training were set as follows: epoch = 80, batch-size = 12, optimizer = SGD, initial learning rate (lr) = 0.01, and momentum = 0.937.

### 3.1. Dataset

Datasets play a crucial role in testing the performance of an algorithm model. However, currently, there is a scarcity of publicly available and well-annotated flame datasets. To address this issue and construct a large-scale dataset with abundant flame samples, we collected flame images from various sources. Additionally, images with flame-like objects were also collected as negative samples to reduce the false alarm rate of the model. By incorporating a diverse range of flame and non-flame images, the aim was to enhance the generalization capability of our model.

**Figure 5:**
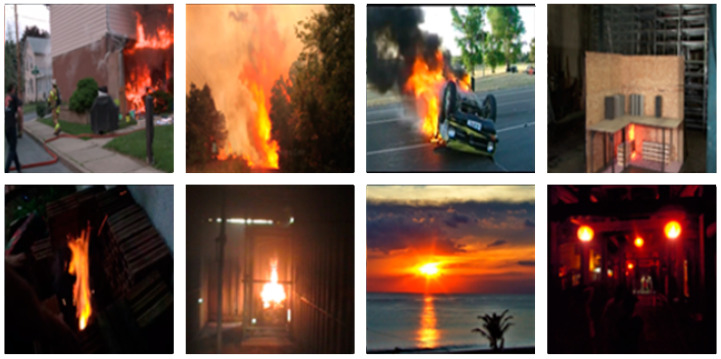
Example samples in data set.

The main sources of the dataset include the BoWFire dataset [19], KMU Fire dataset [20], and a custom-made dataset. The custom-made dataset consists of various types of fire videos, including firefighting experiments, natural fire incidents, fire images from the Internet, and artificially created fire videos. For the video data, images are saved every 15 frames. The final flame dataset contains a total of 9742 images, including 8149 flame images and 1593 non-flame images. The dataset covers different scenes such as indoors, outdoors, buildings, forests, highways, and factories, under different weather and lighting conditions. Because the images are collected from different sources, the original resolutions are also very different, varying from small sizes such as 110 × 185 and 200 × 150, etc., to large sizes such as 1920 × 1080 and 6000 × 4000, etc., which are all resized into 640 × 640 during training. Some samples from the dataset are shown in Figure 5. The LabelImg annotation tool was used to annotate each image in the collected dataset.

### 3.2. Evaluation Methods

To evaluate the performance of the proposed method, we use the following evaluation metrics: precision (P), recall (R), F-measure (FM), average precision (AP), and frame rate (FR). Precision measures the ability of the model to correctly predict positive samples among all the predicted positive samples. Recall measures the ability of the model to correctly predict positive samples among all the actual positive samples. FM is the weighted harmonic mean of Precision and Recall. AP represents the area under the Precision–Recall curve, reflecting the model’s ability to make correct judgments. FR reflects the inference speed of the model. AP and FM are comprehensive performance indicators, and a higher value indicates better model performance. The definitions of P, R, and FM are as follows:

(6) $$P=\frac{\mathrm{TP}}{\mathrm{TP}+\mathrm{FP}}$$

(7) $$R=\frac{\mathrm{TP}}{\mathrm{TP}+\mathrm{FN}}$$

(8) $$\mathrm{FM}=\frac{2\mathrm{PR}}{P+R}$$

where TP represents true positives (the number of samples correctly predicted as positive), FP represents false positives (the number of samples incorrectly predicted as positive), and FN represents false negatives (the number of samples incorrectly predicted as negative). AP is the average precision calculated based on the model’s prediction results. FR is the frame rate, which reflects the inference speed of the model.

### 3.3. Ablation Experiments

To demonstrate the rationality and effectiveness of the proposed improvements, a series of ablation experiments were conducted on our dataset. The YOLOv5s network was used as the baseline model, and three different modifications were introduced: the DBS module, Focal Loss, and EIOU Loss. The results of the experiments are shown in Table 1. For each evaluation metric, the best result is marked in bold.

According to the result data in the table, it can be observed that, by improving the network structure in the YOLOv5s model with the introduction of the DBS module, the precision (P) of flame detection obtained an increase of 2.6%. After incorporating Focal Loss and EIOU Loss, the precision (P) further had an improvement of 5.1%. Ultimately, the proposed method achieved a 6.2% increase in precision (P) for flame detection. Generally, an increase in precision (P) may result in a slight decrease in recall (R). In such cases, it is important to focus on comprehensive performance metrics such as F-measure (FM) and average precision (AP). It can be observed that, under the corresponding improvement strategies, FM achieved improvements of 0.3%, 2.2%, and 3%, and AP obtained increases of 0.4%, 1.2%, and 1.7%, respectively.

The results indicate that the proposed method, which combines the characteristics of flame objects with the improvements made to the YOLOv5s network, is effective in enhancing the flame detection performance.

### 3.4. Method Comparison Experiments

In order to further demonstrate the superiority of the proposed method, several representative object detection network models were selected for comparative experiments. The compared models include RetinaNet, SSD_MobileNet, Faster RCNN, YOLOv5s, YOLOv7-tiny, YOLOv8-n, and YOLOv8-s. Except for Faster RCNN, which is a two-stage object detection network, the rest are lightweight, single-stage object detection networks. 

**Figure 6:**
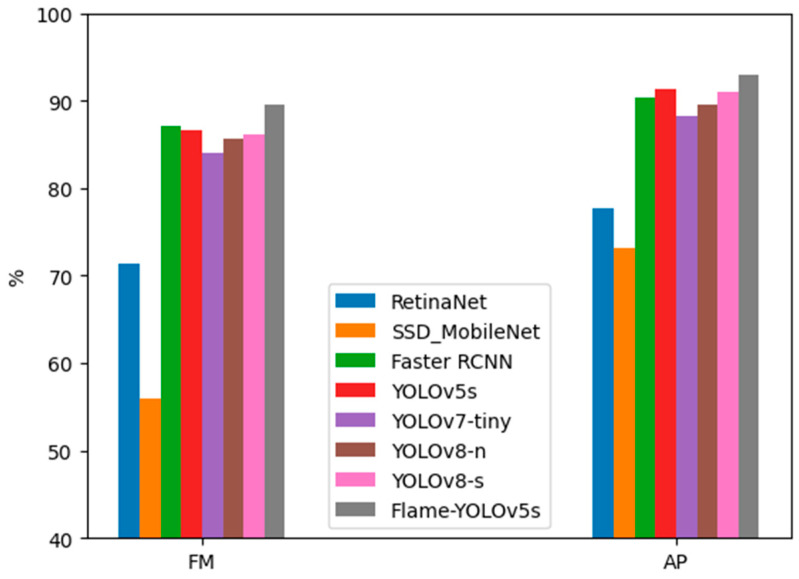
The corresponding bar charts of FM and AP in Table 2.

The experimental results are shown in Table 2, where the best result is marked in bold and the listed frame rate (FR) represents the range of the inference speed on the test dataset images. The corresponding bar charts of FM and AP are also depicted in Figure 6.

From Table 2 and Figure 6, it can be observed that Faster RCNN, which has the advantages of a two-stage object detection network, indeed performs better than lightweight single-stage object detection networks like RetinaNet, SSD_MobileNet, YOLOv7-tiny, YOLOv8-n, and YOLOv8-s. However, due to its non-lightweight design, it has a relatively lower detection speed of only 13–17 FPS. YOLOv5s shows a comparable performance to Faster RCNN while achieving a higher detection speed of 25–27 FPS. Flame-YOLOv5s, which is our proposed method in this paper, achieves the highest precision (P) among all the models, with a recall rate (R) slightly lower than that of the original YOLOv5s. However, it outperforms the other models in terms of the comprehensive evaluation metrics FM and AP, achieving the best overall performance. Its detection speed is also capable of reaching 24–26 FPS, meeting the requirements for real-time detection.

### 3.5. Example Demonstrating the Effectiveness of Flame Detection

**Figure 7:**
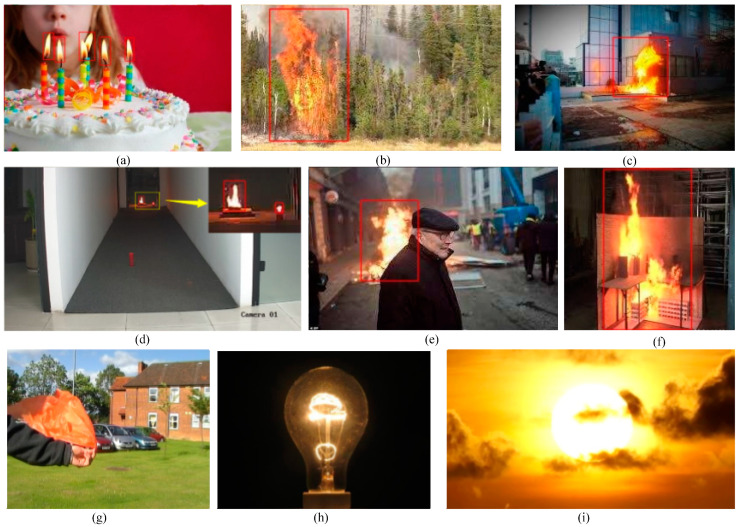
Detection results of Flame-YOLOv5s on real-world test images.

To visualize the detection performance of the Flame-YOLOv5s network on flame objects and flame-like objects, we selected a subset of representative test images from the dataset. These images depict various real-world scenarios, including indoor and outdoor scenes, flame objects of different scales, and some flame-like objects. The detection results are shown in Figure 7, where the detected flame objects are highlighted with rectangular bounding boxes. It is evident that Flame-YOLOv5s successfully detects flame objects of various scales in the different scenes depicted in rows 1 and 2 of Figure 7a–f. In particular, small-sized ignited candles can be observed in Figure 7a,d. Regarding the unignited red candle in Figure 7d, the red scarf, the illuminated light bulb, and the sunset and clouds that resemble flame-like objects in row 3 (Figure 7g–i), Flame-YOLOv5s does not mistakenly classify them as flames. This demonstrates that Flame-YOLOv5s exhibits a good robustness against strong distractions of this nature.

**Figure 8:**
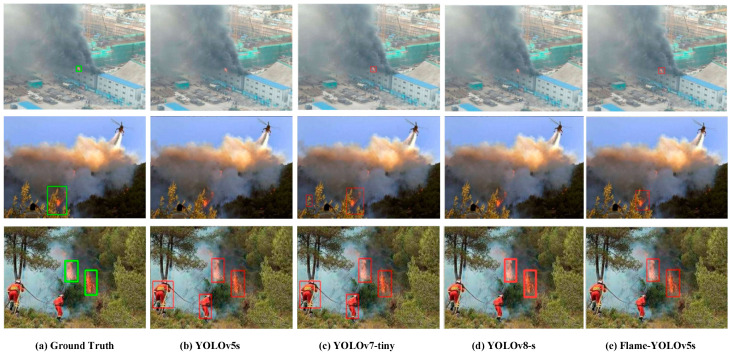
Comparison of detection results of Flame-YOLOv5s with other typical lightweight YOLO based methods.

In addition, Figure 8 shows the comparison results of the proposed method with the ground truth and other typical lightweight YOLO-based methods, including YOLOv5s, YOLOv7-tiny, and YOLOv8-s. In row 1, only YOLOv7-tiny and Flame-YOLOv5s detect the small flame object; in row 2, only Flame-YOLOv5s obtains the right result, YOLOv7-tiny has a mistaken result, and the other two methods cannot detect the flame; and in row 3, YOLOv5s and YOLOv7-tiny classify the two firefighters in red uniform as flames mistakenly, and YOLOv8-s and Flame-YOLOv5s detect the flames accurately.

**Figure 9:**
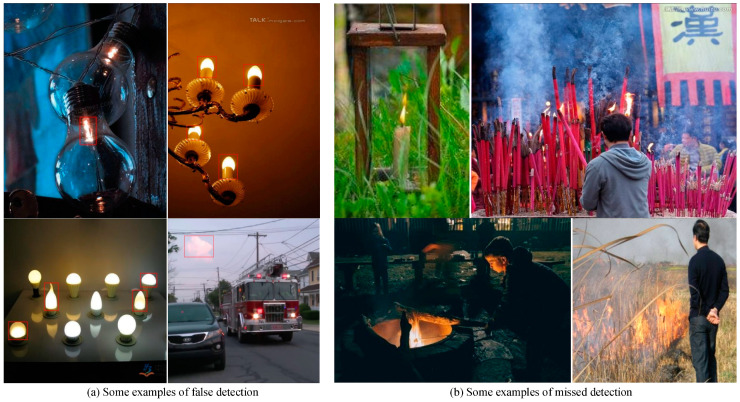
Some examples of false detection and missed detection by the proposed Flame-YOLOv5s.

Although an improved performance was obtained by Flame-YOLOv5s, it is inevitable that there are some false detection and missed detection results. Some examples are shown in Figure 9. In Figure 9a, the false detection of flame objects may be caused by occasional high confidence in flame-like objects such as electric lamps or clouds with a very similar shape and color to a flame. In Figure 9b, the missed detection of flame objects may occur due to flame disturbance deformation or partial occlusion. To deal with these limitations of Flame-YOLOv5s in single-frame detection mode, we use the time sequence analysis strategy to alleviate the false alarms in the video detection mode from the practical application point of view.

### 3.6. The Impact of Time Sequence Analysis Strategy on Video Flame Detection

To demonstrate the effectiveness of the time sequence analysis strategy, an experimental study was conducted using 40 flame videos and 10 flame-like videos. The experimental results are presented in Table 3. In the table, “Continuous Detection” indicates the absence of the time sequence analysis strategy, where the alarm is triggered when a frame with a flame object is detected in the video. The alarm continues as long as subsequent frames with flame objects are detected, and it ends when no flame object is detected in a frame, counting as one alarm and waiting for the next alarm. The number of alarms using the time sequence analysis strategy is determined and indicated in the table based on the method described in Section 2.3 with the parameter setting K=100,τ=80%.

According to the data analysis from Table 3, it can be observed that, after employing the time sequence analysis strategy, the short-term disturbances caused by flame perturbation, flowing occlusions, and interference caused by flame-like objects do not affect the continuous alarms. Throughout the experimental testing process, the frequency of alarms decreases, while the duration of each alarm increases.

## 4. Conclusions

The real-time detection of flame videos has been a pressing technical challenge in the field of firefighting in recent years. In this paper, the Flame-YOLOv5s network was proposed by introducing deformable convolutions to improve the network architecture. It enhanced the feature extraction capability for irregular flames while considering the detection speed. The use of Focal Loss as the classification loss function addressed the issues of imbalance between positive and negative samples of flames and backgrounds, as well as the imbalance between easy and hard samples. The adoption of EIOU Loss as the regression loss function resolved the problems of slow network training convergence and inaccurate regression positioning. By employing a time sequence analysis strategy, this reduced false alarms caused by flame shape variations, flame occlusion, and flame-like object interference. During the network training, flame-like images were included as interference items to ensure the generalization ability and stability of our algorithm model. The proposed method achieved satisfactory results in terms of accuracy and real-time performance, demonstrating its practical value and potential for application in the field of fire safety monitoring. The current method only detects flame objects and does not consider smoldering phenomena. Therefore, in future research, we will add a smoke detection module to achieve more timely prediction and early warning of fires, serving a preventive role.

## Figures and Tables

**Table 1:** Results of ablation experiments (%).

DBS	Focal Loss + EIOU Loss	P	R	FM	AP
×	×	84.0	**89.3**	86.6	91.3
√	×	86.6	87.3	86.9	91.7
×	√	89.1	88.6	88.8	92.5
√	√	**90.2**	89.1	**89.6**	**93.0**

**Table 2:** Performance comparison of different models on self-made flame data set.

Model	P/%	R/%	FM/%	AP/%	FR/FPS
RetinaNet	63.3	81.9	71.4	77.7	28~31
SSD_MobileNet	42.0	83.8	55.9	73.2	32~34
Faster RCNN	85.6	88.9	87.2	90.3	13~17
YOLOv5s	84.0	**89.3**	86.6	91.3	25~27
YOLOv7-tiny	82.2	85.8	84.0	88.3	26~29
YOLOv8-n	88.2	83.2	85.7	89.5	42~45
YOLOv8-s	87.7	84.5	86.1	91.0	33~37
Flame-YOLOv5s	**90.2**	89.1	**89.6**	**93.0**	24~26

**Table 3:** Comparison of alarm frequency with and without using the strategy of time sequence analysis.

Video Type	Alarm Times for Continuous Detection	Alarm Times for Time Sequence Analysis
Video with Flame Objects	103	47
Video with Flame-like Objects	36	0

## Data Availability

The data used to support the findings of this study are available from the corresponding author upon reasonable request.
